# Transcriptome Analysis of Natural Killer Cells in Response to Newcastle Disease Virus Infected Hepatocellular Carcinoma Cells

**DOI:** 10.3390/genes14040888

**Published:** 2023-04-10

**Authors:** Juanjuan Huang, Tingting Zheng, Ying Liang, Ying Qin, Xing Wu, Xiaohui Fan

**Affiliations:** 1 State Key Laboratory of Targeting Oncology, National Center for International Research of Bio-Targeting Theranostics, Guangxi Key Laboratory of Bio-Targeting Theranostics, Collaborative Innovation Center for Targeting Tumor Diagnosis and Therapy, Guangxi Medical University, Nanning 530021, China; 2 Department of Microbiology, School of Preclinical Medicine, Guangxi Medical University, Nanning 530021, China; 3 Key Laboratory of Basic Research on Regional Disease, Education Department of Guangxi, Guangxi Medical University, Nanning 530021, China

**Keywords:** Newcastle disease virus, natural killer cell, hepatocellular carcinoma, immunity, cancer therapy

## Abstract

When tumor cells are infected by the Newcastle disease virus (NDV), the lysis of tumor cells by natural killer (NK) cells is enhanced, which may be related to the enhanced NK cell activation effect. To better understand the intracellular molecular mechanisms involved in NK cell activation, the transcriptome profiles of NK cells stimulated by NDV-infected hepatocellular carcinoma (HCC) cells (NDV group) and control (NC group, NK cells stimulated by HCC cells) were analyzed. In total, we identified 1568 differentially expressed genes (DEGs) in the NK cells of the NDV group compared to the control, including 1389 upregulated and 179 downregulated genes. Functional analysis showed that DEGs were enriched in the immune system, signal transmission, cell growth, cell death, and cancer pathways. Notably, 9 genes from the IFN family were specifically increased in NK cells upon NDV infection and identified as potential prognosis markers for patients with HCC. A qRT-PCR experiment was used to confirm the differential expression of IFNG and the other 8 important genes. The results of this study will improve our understanding of the molecular mechanisms of NK cell activation.

## 1. Introduction

Newcastle disease virus (NDV) is a single-strand negative-sense RNA virus that can affect more than 250 bird species [1]. Despite the fact that NDV causes respiratory, neurologic, and enteric diseases in birds, it is mostly asymptomatic in humans [2]. Decades of research have demonstrated that NDV has oncolytic properties as it can selectively kill tumor cells [3,4]. In 1964, Wheelock and Dingle first reported that NDV significantly reduced the number of tumor cells in leukemia patients [5]. In 1965, Cassel and Garrett confirmed that intratumoral injection of NDV oncolytic strain 73-T can cause tumor regression in cervical cancer patients [6]. Subsequently, the applications of NDV in clinical treatment for tumor regression and in vivo experiments in mice were successively reported [7,8,9,10,11]. For example, autologous NDV-modified tumor cells can effectively treat colorectal cancer patients with liver metastases [7,8]. The results of clinical treatment with NDV in 83 stage III melanoma patients showed that the tumor in the patients was effectively suppressed and the survival of the patients was significantly prolonged [9]. Lorence et al. confirmed that local injection of the NDV 73-T strain can completely lyse the tumor in nude mice that were transplanted with human fibrosarcoma [10]. Li et al. reported that the NDV F3aa strain can effectively inhibit the proliferation of head and neck cancer in tumor-bearing mice [11]. However, the molecular mechanisms behind the oncolytic properties of NDV are largely unknown.

Several pathways have been reported to be modulated by NDV in tumor cells. The LaSota strain of NDV can selectively bind to the membrane of human tumor cells and enter the cytoplasm via endocytosis, resulting in apoptosis and DNA fragmentation of tumor cells [12]. In NDV-treated mouse tumors, the expression levels of P21, P27, and P53 genes were increased, while the expression levels of CD34, integrin α 5, VEGF, and VEGF-R genes were decreased [12]. ROS production and apoptosis pathways were reported to be stimulated by NDV in these mouse models. In addition, NDV can induce apoptosis via caspase-dependent and caspase-independent pathways, and Deng et al. reported that NDV can downregulate the expression of phosphoglycerate kinase (PGK) genes, which encode glycolytic enzymes that function in the glycolytic pathway, in the NDV-infected peripheral blood mononuclear cells (PBMCs) [13,14]. In addition, IFN and RAS signaling pathways have also been reported to be affected by NDV in tumor cells [15,16,17,18,19]. On the other hand, NDV activates a non-specific anti-tumor immune response through the secretion of cytokines, including IFN-α, IFN-β, TNF-α, and IL-1, which directly activate NK cells and macrophages [20,21,22]. Fournier et al. [19] reported that the envelope protein (HN) and viral double-strand RNAs of NDV can activate the IFN response [23,24]. BHK cells transfected with the NDV-HN gene can induce the human PBMC to produce IFN-α and induce the expression of TRAIL in CD14^+^ monocytes and CD3^+^ T cells [25]. Further, Yusoff et al. confirmed that NDV can enhance the anti-tumor activity of human NK cells in vitro [26]. NK cells stimulated by NDV in vitro were infused into mice with breast tumor. IL-2, TNF-α, and IFN were secreted by NDV-activated NK cells. In our previous studies, we also found that the upregulation of the TRAIL gene might be related to the enhanced cytotoxic effect of NK cells stimulated by NDV [27,28,29]. However, much is unknown about the gene changes in NK cells stimulated by NDV.

Numerous studies have demonstrated that NK cells have an enhanced lysis effect on NDV-infected tumor cells [27,30]. The possible mechanisms may include the expression of HN protein in NDV-infected tumor cells, enhanced adhesion with NK cells, the release of more NK cell response cytokines (e.g., IFN-γ, TNF-α, perforin, and granzyme B), and the activation of apoptosis in tumor cells. However, the intracellular molecular mechanisms of NK cell activation by NDV are not clear. In the present study, we performed transcriptome sequencing for the NK cells upon the stimulation of NDV-infected hepatocellular carcinoma (HCC) cells. Bioinformatics analyses were used to identify genes in NK cells in response to NDV. Functional analysis revealed NK cells and immune-related pathways were activated by the NDV. This is the first transcriptome study of NK cells stimulated by NDV-infected HCC cells. The results will improve our understanding of NK cell activation and molecular mechanisms upon NDV infection.

## 2. Materials and Methods

### 2.1. Cell Preparation

The NDV strain 7793 was provided by the Department of Microbiology, School of Preclinical Medicine, Guangxi Medical University (Nanning, China), and maintained under laboratory conditions. Human NK92MI cells, an interleukin-2 (IL-2)-independent cell line derived from NK-92 cells with superior cytotoxicity to a wide range of tumor cells, were purchased from the American Type Culture Collection (ATCC, Manassas, VA, USA), and the human hepatoma Huh7 cells were obtained from the cell bank of the Chinese Academy of Sciences. We first resuspended the Huh7 cells in serum-free DMEM medium at a concentration of 5 × 10^5^ in six-well plates with 1 mL per well, as we found that cell culture with serum had an impact on the virus duplication in our pre-experiments. For the NDV group, 0.1 HU/mL NDV was added to the Huh7 cells and co-cultured at 37 °C for 16 h. Simultaneously, the negative control (NC) group was added with PBS. Each group was subsequently irradiated with ultraviolet light for 5 min and the supernatant was discarded. Then, NK cells suspension was added to each group at an E/T ratio of 1:1. After 24 h, the cell culture medium was collected and incubated for 3 min. After half of the top medium was discarded, we collected and centrifuged the NK cells under 1000× *g* at 4 °C for 10 min. To preserve the RNA integrity, TRIzol reagent (Invitrogen, Carlsbad, CA, USA) was added to the NK cells as fast as possible, and the samples were stored at −80 °C.

### 2.2. Real-Time Cell Analysis

We used 2 × 10^4^ Huh7 cells/well as starting material to perform the real-time cell analysis. Initially, the Huh7 cells were inoculated onto E-plate 16 (Agilent Technologies, Santa Clara, CA) plates for 24 h. Half of the wells were used as negative controls (Huh7 cells), and the other half of the wells were added with 0.1 HU/mL NDV (NDV + Huh7 cells) and incubated for 16 h. Then, all the cells were irradiated by UV for 5 min, and the supernatant was discarded. Half wells of the Huh7 and NDV + Huh7 groups were added with NK cells (E/T = 1:1) and used as the NK (Huh7 + NK cells) and NDV + NK (NDV + Huh7 + NK cells) groups, respectively. Then, during the 24 h period of cell culture, the cell proliferation rates were analyzed using the xCELLigence Real-Time Cell Analysis (RTCA) DP instrument (Agilent Technologies, Santa Clara, CA, USA). Cell counts at 6 h, 12 h, and 24 h were used for statistical analysis. Triplicates were performed for the RTCA.

### 2.3. RNA Isolation, Library Construction, and Sequencing

The total RNA was isolated from six samples in two groups using TRIzol reagent according to the manufacturer’s instructions. RNA concentration and purity were determined by the NanoDrop 8000 (Thermo Fisher Scientific, Waltham, MA, USA) system. Then, the RNA integrity was evaluated using the Agilent 2100 Bioanalyzer with the RNA 6000 Nano Kit (Agilent Technologies, Santa Clara, CA, USA). Samples with an RNA integrity number (RIN) ≥7 were utilized for subsequent experiments. The mRNA libraries were constructed at the Beijing Genomics Institute (BGI) (Wuhan, China) and sequenced on the BGISEQ-500 platform, as previously described [31], with a paired-end 150 bp (PE150) strategy.

### 2.4. Analysis of RNA-Seq Data

The raw sequencing reads were processed for data cleaning using SOAPnuke [32], according to the following criteria: (i) removing the adapter reads; (ii) removing reads if N bases accounted for more than 5% of the read; (iii) removing reads if bases with a quality lower than 15 were more than 20% of the read. Then, we mapped the clean reads to the human reference genome (GRCh37/hg19) and transcript database using HISAT2 and Bowtie2 (v2.2.5), respectively [33,34]. RSEM was used to calculate the gene expression profiles of all six samples, and the FPKM (fragments per kilobase of transcript per million fragments) method was used for normalization [35]. DESeq2 was used to identify the DEGs between the NDV and NC groups using the following filter criteria [36]: log2|fold change| > 1 and q-value < 0.05.

### 2.5. Gene Ontology and Pathway Enrichment Analysis

Functional analysis of the DEGs was performed using the GO and KEGG databases [37,38]. The statistical significance of enriched GO terms and pathways by the DEGs was assessed by a hypergeometric test and corrected by the Benjamini–Yekutieli method at FDR (false discovery rate, <0.05). GO and KEGG pathway enrichment analysis was performed and visualized using the OmicShare tools (https://www.omicshare.com/, accessed date: 5 December 2022). *p*-values and q-values were calculated to show the significance of gene enrichment and to adjust the *p*-values, respectively, as described [39]. The chord plot for selected signaling pathways was drawn using the GOplot package [40].

### 2.6. Survival Analysis

Kaplan-Meier Plotter (https://kmplot.com/analysis/ accessed date (5 December 2022)) is a publicly accessible database that provides gene expression data to assess the survival in patients from multiple cancer types, including liver cancer. In this study, gene expression data and survival information of 364 hepatocellular carcinoma patients were downloaded from the TCGA and GEO databases [41]. We selected 17 DEGs related to the NK cell-mediated cytotoxicity pathway for survival analysis between patients with low and high expression. Statistical significance was determined by the hazard ratio (HR) with a 95% confidence interval (CI) and log-rank *p*-value.

### 2.7. Quantitative Real-Time RT-PCR (qRT-PCR)

In order to verify the reliability of the transcriptome sequencing analyses, nine candidate genes associated with NK cell activation were selected, and GAPDH was used as the internal control for qRT-PCR. Total RNA (500 ng~2 ug) was first synthesized to cDNA using the reverse transcriptase kit (Invitrogen). Then, cDNA was used as templates for qRT-PCR using the SYBR Premix Ex Taq II Kit (Takara, Dalian, China), according to the manufacturer’s protocol. The experiments were conducted using a CFX96 Touch real-time PCR system (Bio-Rad Laboratories, Hercules, CA, USA) with the following program: 95 °C for 10 min, 40 cycles of 95 °C for 10 s, and 60 °C for 60 s. Then, the melting curve was run from 60–95 °C with each amplification for three replicates. The relative gene expression was analyzed using the 2^−ΔΔCt^ method [42]. Primers were synthesized by Shanghai Sangon Bio-technology Co. (China) and can be seen in Table S1.

## 3. Results

### 3.1. Summary of Transcriptome Sequencing

**Figure 1:**
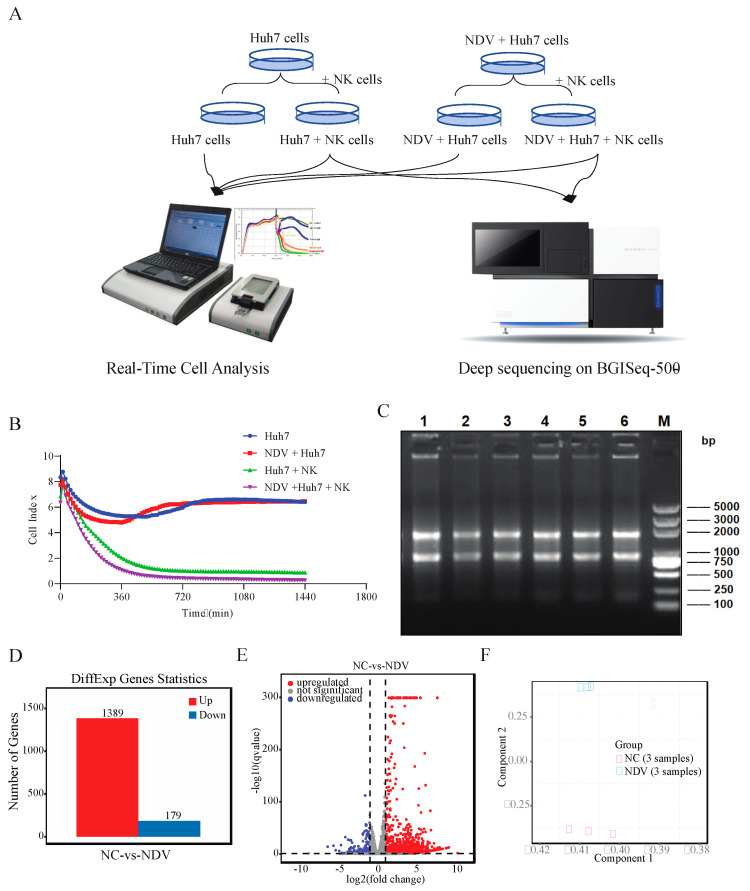
Study design and identification of DEGs between NDV and NC groups. (**A**) Experiment design for this study. (**B**) RTCA results of the Huh7 cells with different treatments. (**C**) Electropherograms of total RNAs isolated from the samples for deep sequencing. (**D**) Numbers of upregulated and downregulated genes identified in the NDV group compared to NC group. (**E**) Volcano plot displaying DEGs between NDV and NC groups. X-axis displays the value of log2 fold change, and the y-axis shows the -log10 (q-value). Upregulated genes are displayed by the red dots, downregulated genes are displayed by the blue dots, and the grey dots represent genes with no significant changes between NDV and NC groups. (**F**) PCA plot of DEGs. A dot represents a single sample, each group is represented by three replicates.

We showed in Figure 1A the study design for the investigation of the immunotherapy effects of NK cells on NDV-infected hepatoma cells. First, we performed the real-time cell analysis for cells from the NC (Huh7 + NK cells) and NDV (NDV + Huh7 + NK cells) groups, together with cells under another two conditions—Huh7 cells and NDV + Huh7 cells. It was clear that NK cells have the capacity to kill hepatoma cells (*p* < 0.0001) and that NDV has a very limited impact on the cell death of Huh7 cells (Figure 1B). However, when NDV-infected Huh7 cells were treated with NK cells, the NK-mediated cell lysis was significantly enhanced (*p* < 0.05), compared with the NC group (Figure 1B).

Additionally, to understand the possible molecular mechanism behind the enhanced immunotherapy effect of NK cells in NDV infected hepatoma cells, we purified the NK cells from the NC and NDV groups and isolated total RNA from these NK cells. After the quality and quantity of the total RNA were evaluated by the Agilent Bioanalyzer (Figure 1C), they were sequenced on the BGISeq-500 platform (Figure 1A). The summary of sequencing characteristics and genome mapping results are described in Table 1. After low quality reads were filtered out, we obtained 266 million clean reads for all six libraries, with a range of 44.21 million to 45.17 million reads per sample. It showed that more than 97% of the clean reads had a quality score of Q20 (a base quality of >20 and an error rate <0.01). Then, we found 93.40–94.90% of the clean reads mapped to the human reference genome with a high unique mapping ratio (86.21–87.48%). Whereas the gene alignment rates were generally lower than the genome alignment rates, the six samples displayed 76.51–78.46% for overall gene mapping and 72.07–74.21% for unique gene mapping. These data indicated that the sequencing outcome was sufficient for further analysis of mRNA between the NC and NDV groups.

### 3.2. Identifcation of DEGs bewteen NC and NDV

Next, we profiled the gene expression for the samples in the NC and NDV groups based on the gene alignment results. Among the 16,707 detected genes in the six libraries (Table S2), we found 9952 and 10,139 genes (FPKM > 1) in the NC and NDV groups, respectively. Interestingly, according to the average FPKM values, nine (e.g., RPS27, TMSB4X, RPS2, NKG7, IL32, ACTG1, RPL30, RPS11, and GAPDH) of the top 10 highly expressed genes were shared by the NDV and NC groups. Then, we used DEseq2 to identify DEGs between NDV and NC groups, which might be related to the NDV effects on the NK cells. Using the criteria described above, 1568 significant DEGs, including 1389 upregulated genes and 179 downregulated genes, were identified (Figure 1D,E, and Table S3). According to the log2FC values, the top 5 upregulated genes were H2AC19, KLF11, CREB5, IFNB1, and NEURL3, and the top 5 downregulated genes were ITIH2, LDLRAD2, SERPIND1, PCSK9, and A2M (Table S3). In addition, the principal-component analysis (PCA) confirmed the separation of NC and NDV samples (Figure 1F) and supported the repeatability of biological replicates within each group.

### 3.3. Gene Ontology Enrichment Analysis of DEGs

**Figure 2:**
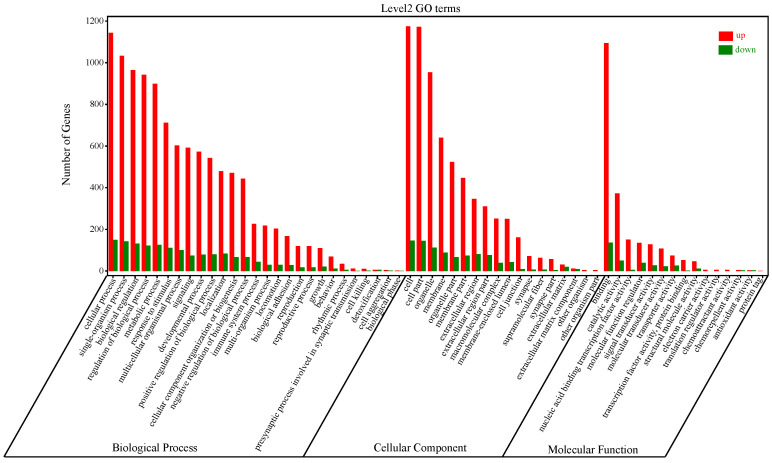
GO analysis of the 1568 DEGs at level 2. Genes were classified into biological processes, cellular components, and molecular functions. The y-axis shows the number of genes in each category. The red bars represent up-regulated genes, and green bars represent down-regulated genes.

Next, we analyzed the gene ontologies enriched by the 1568 DEGs. As shown in Figure 2 and Table S4, 8234, 786, and 1587 GO terms were assigned to biological process, molecular function, and cellular component, respectively. Notably, we found 1294, 1095, 1175, 1025, 270, and 13 DEGs enriched in the biological processes of cellular process, biological regulation, single-organism process, metabolic process, immune system process, and cell killing. The upregulation of these DEGs indicated that they might promote NK cell adhesion, activation, and the immune response. For cellular component annotation, most of the DEGs were evenly distributed in cell components, including organelles, cell membranes, membrane-enclosed lumen, extracellular regions, cell associations, junctions, and synapse. Molecular function enrichment analysis showed that DEGs in NDV-stimulated NK cells were associated with binding, catalytic activity, molecular function regulation, molecular transduction, transport, and molecular structure.

### 3.4. KEGG Pathway Enrichment Analysis of DEGs

**Figure 3:**
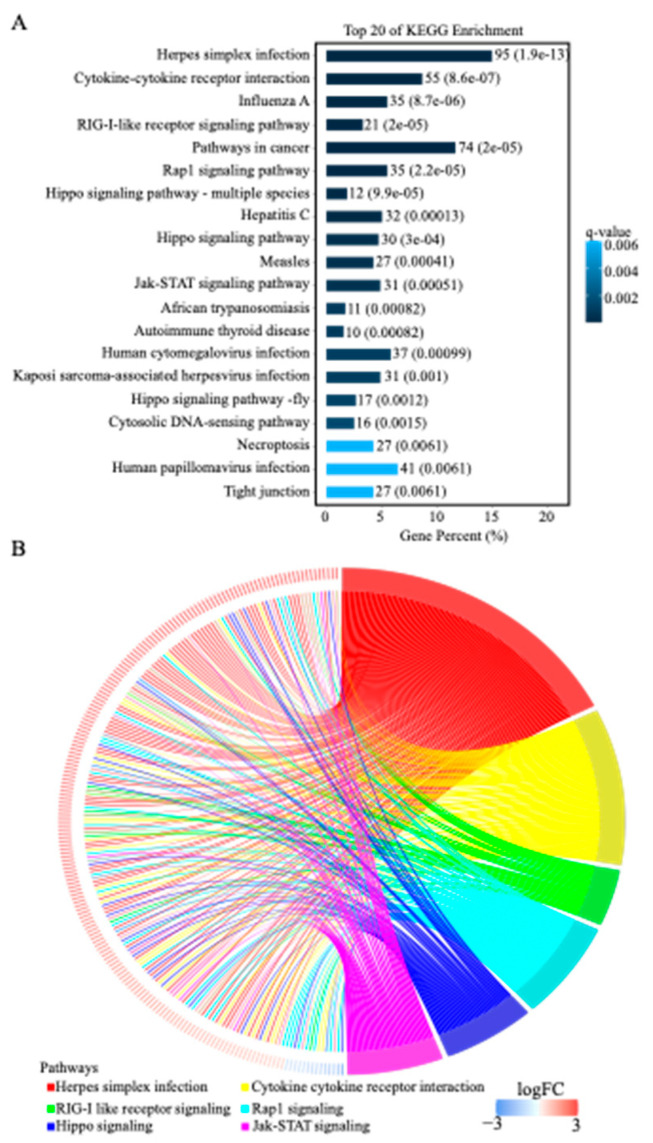
Significant KEGG pathways enriched by the DEGs. (**A**) Top 20 significant KEGG pathways enriched by the DEGs. The x-axis represents the proportion of annotated genes of pathways to all annotated DEGs (total 632 genes). (**B**) Chord plot of selected six signaling pathways. Scale bar represents the log2FC values of DEGs on the left half of the outer circle.

Next, KEGG pathway enrichment analysis was performed to identify possible pathways involving the DEGs in the NDV-stimulated NK cells. A total of 304 KEGG pathways were found for the 1568 DEGs, and 44 of them were significantly enriched (q-value < 0.05) (Table S5). We showed in Figure 3A the top 20 significant KEGG pathways enriched by the DEGs, and most of them were associated with human cancer, such as cytokine-cytokine receptor interaction, RIG-I-like receptor signaling pathway, pathways in cancer, rap1 signaling pathway, and Jak-STAT signaling pathway. Notably, we found that the genes involved in immune system-related pathways (e.g., RIG-I-like receptor signaling pathway, natural killer cell-mediated cytotoxicity) were upregulated in NK cells upon NDV infection. In addition, we selected 6 pathways (202 DEGs) and visualized them using a chord plot (Figure 3B). It showed that most (188/202, 93.07%) of the genes involved in these pathways were upregulated, and that Rap1 signaling had the most (7/14, 50%) downregulated genes among them.

### 3.5. Identification of Genes Related to NK Cell Activation

In order to further investigate the mechanisms underlying the promotion of NK cell activity by NDV-infected hepatoma cells, we manually screened the 26 important pathways related to NK cell activation (Table 2). According to the classification rules of KEGG, these pathways are mainly distributed among four KEGG functions: immune system (9 pathways), cancer (2 pathways), signal transduction (13 pathways), and cell growth and death (2 pathways). These results indicated that the activation mechanisms of NK cells might be related to the activation of signal transduction and immune factors. Interestingly, the signal pathway of hepatocellular carcinoma was also significantly enriched, which may be related to the specific induction by NDV. We further analyzed the genes involved in these pathways, and a total of 261 unique genes were identified (Table S6). Among them, a considerable number of genes were involved in the regulation of multiple pathways, such as TRAIL (TNFSF10), FAS, IFNG, and IL10. These genes related to NK cell activation, identified for the first time in this study, can provide important references for future studies.

### 3.6. Kaplan-Meier(K-M) Survival Analysis

**Figure 4:**
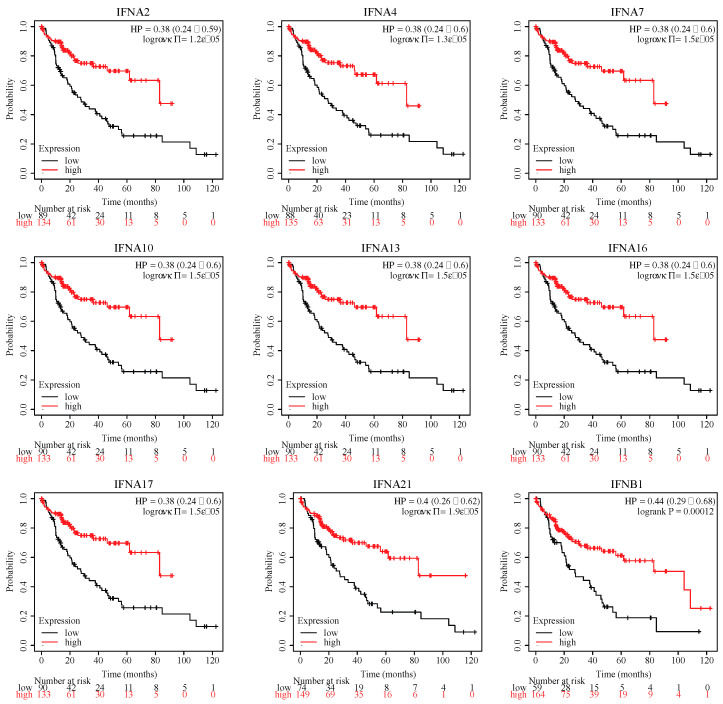
The Kaplan-Meier curves for nine IFN genes. The abscissa represented the survival time of patients with HCC, and the ordinate represented survival rate of patients with HCC. HR of 1 indicates no effect on risk; HR between 0 and 1, good prognosis; HR greater than 1, poor prognosis.

In addition, to further validate the genes related to the NK cell-mediated cytotoxicity pathway (ko04650), we performed a Kaplan-Meier overall survival analysis of 364 patients with HCC for 17 DEGs of interest. Interestingly, all 17 of these genes were upregulated in the NDV group compared to the NC group. By setting HR > 1 and downregulation or HR < 1 and upregulation as the screening conditions, 9 genes, including 8 IFNA family members and 1 IFNB family member, were significantly related to the prognosis of liver cancer (Table S7, Figure 4). As shown in Figure 4, the HR values for these IFN family DEGs were below 0.5 in liver cancer patients with logrank (*p*) < 0.001. This result shows the patients with HCC had a better overall survival rate (OS) with high expression of INF family genes (HR < 1, *p* < 0.05), suggesting that the high expression of IFN family genes may be related to the prolongation of survival in patients with liver cancer. It is also worth noting that although PAK1 and IFNGR2 satisfy the conditions of HR > 1 and *p* < 0.05, they are upregulated genes and cannot be considered as tumor risk factors.

### 3.7. Validation of Gene Expression by Real-Time PCR

**Figure 5:**
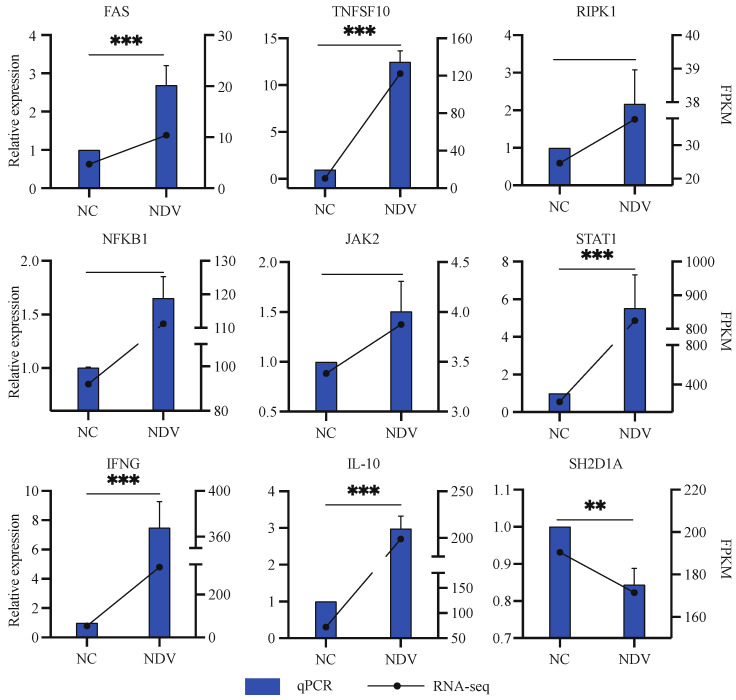
qPCR validation (*n* = 3). ** and *** indicate significant differences at q < 0.01 and q < 0.001, respectively.

We selected 9 NK cell activation-associated DEGs, including 8 upregulated genes (FAS, TNFSF10, RIPK1, NFKB1, JAK2, STAT1, IFNG, and IL-10), and one downregulated gene (SH2D1A), and performed a qPCR experiment for validation. GAPDH was used as an internal control. Triplicates were performed for each gene in each sample, and ΔΔCt method was used to show the gene expression. Interestingly, the expression patterns of all 9 genes were agreed upon by qRT-PCR and transcriptome sequencing (Figure 5). This indicated that the genes identified in this study might be associated with NK cell activation by NDV.

## 4. Discussion

In the present study, we analyzed the transcriptome profiles of NK cells stimulated by NDV-infected Huh7 cells and identified 1568 DEGs that might be related to NK cell activation and killing activity. Among the DEGs, some previously reported NK cell immunity-related genes were identified (Table S3), such as TRAIL (TNFSF10), FAS, IL10, and IFNG. Cellular cytotoxicity is an important mechanism of the immune system against viral infection and is mainly mediated by the cytotoxic T cells and NK cells [43]. It is generally assumed that NK cells can kill virus-infected transformed cells by directly releasing perforin and granzyme B or by Fas- and TRAIL-induced death receptor-mediated apoptosis [27,44,45]. TARIL, FAS, and TNF, as death receptors, play important roles in the process of apoptosis [46,47]. TRAIL strongly induces apoptosis in transformed cancer cells and causes no observed negative side effects for the host cells. It displays considerable antitumor activity in xenograft models, including colon, breast, multiple myeloma, glioma, and prostate cancers [48]. In the current study, the expression of TRAIL in NK cells was increased by about 10 times upon the stimulation of NDV (Table S3), which is consistent with our previous study [29]. High TRAIL expression may enhance the NK cell induction of tumor cell apoptosis. Similarly, another death receptor, FAS, also showed significant upregulation (Table S3). In addition, some key cytokines, such as IL10 and IFNG, were significantly differentially expressed in the NDV group compared to the NC group. It is notable that although perforin (PRF1) and granzyme B (GZMB), two important oncolytic genes, showed high expression in all six samples, there was no significant difference between the two groups, which may be related to the specific stimulation of NK cells by NDV. To explore the gene associated with NK cell activation upon NDV infection, more HCC cell lines might be applied to reduce the background noise.

Our results showed that pre-treatment of tumor cells with NDV enhanced NK cell activity (Figure 1B), and this might be related to the preparation of the immune microenvironment of the tumor cells. It has been reported that NDV infection creates an inflammatory environment in the tumor microenvironment, which directly activates the NK cells and promotes the recruitment of immune cells [49]. The upregulation of NK cells has been observed in various preclinical studies of recombinant NDV for the treatment of melanoma and lung cancer [49]. In addition, the application of NDV in tumor therapy can enhance T cell recruitment and induce necrosis and pyroptosis of tumor cells [50]. Further, in this study, we found 26 important pathways related to NK cell activation upon NDV infection (Table 2), including 13 signal transduction pathways and nine immune pathways. Notably, RIG-1-like receptors, rap1, hippo, and jak-STAT signaling pathways were upregulated in NK cells upon the NDV infection (Figure 3B). The RIG-I ligand (3pRNA) can directly activate NK cells, and 3pRNA-activated NK cells kill melanoma cells more efficiently than NK cells activated by type I interferon [51]. Rap1b has been reported to facilitate NK cell functions via IQGAP1-mediated signalosomes [52]. Overall, the results may help improve our understanding of the molecular mechanism of NK cell activation and activities upon NDV infection. DEGs and pathways enriched by these DEGs indicate new possible therapeutic targets for cancers, and functional experiments are required to validate their therapeutic potential in HCC.

Furthermore, we identified some potential prognostic biomarkers for HCC, a highly malignant tumor with high incidence and mortality rates, including AFP, H2AC19, KLF11, and IFNG (Table S3, Figure 4). AFP is a well-known biomarker for HCC, and AFP reduction was recently associated with better outcomes among patients with baseline AFP ≥ 400 µg/L or with a history of hepatitis B or C virus or alcohol use [53]. Low KLF11 expression was associated with poor prognosis and poor chemotherapy response in sarcoma patients, and as a TGF-β signaling pathway mediator, KLF11 could be a druggable suppressor for sarcoma cancer stem cells [54]. We examined the prognostic values of 9 IFN genes and found that high expression of IFN genes might be good prognostic biomarkers for HCC (Figure 4). Their prognostic values might be related to the NK cell activities, and more experiments are needed to further validate these results.

## 5. Conclusions

This study provides a comprehensive transcriptome of NK cells stimulated by NDV-infected human hepatoma cells. Compared to NK cells stimulated by tumor cells, we identified 1568 DEGs in NK cells stimulated by NDV-infected tumor cells. In addition, the DEGs are mainly involved in the immune system, signal transmission, cell growth, cell death, and cancer pathways, of which 26 were related to NK cell activation involving 225 upregulated genes. Further, we showed the IFN genes were upregulated in NK cells upon NDV infection, and high expression of IFN genes might be good prognostic biomarkers for HCC. This study provides a molecular basis for NK cell activation upon NDV infection, and the results will benefit future studies in the immunotherapy field. However, these transcriptome data are preliminary and limited, and the function of the DEGs requires further investigation in activated NK cells.

## Figures and Tables

**Table 1:** Overview of the RNA-seq and genome/gene mapping results.

Sample	NC1	NC2	NC3	NDV1	NDV2	NDV3
Raw reads number (M)	45.44	47.19	45.44	45.44	45.44	45.57
Raw bases (G)	6.82	7.08	6.82	6.82	6.82	6.84
Clean reads number (M)	44.39	45.17	44.21	44.24	44.30	44.46
Clean bases (G)	6.66	6.78	6.63	6.64	6.64	6.67
Clean rate (%)	97.68	95.73	97.29	97.37	97.49	97.55
Q20 (%)	97.17	97.37	97.30	97.24	97.36	97.59
Genome mapped rate (%)	94.47	93.66	93.94	93.50	93.40	94.90
Genome uniquely mapped rate (%)	87.23	86.34	86.61	86.32	86.21	87.48
Gene mapped rate (%)	77.43	76.51	76.89	76.07	76.82	78.46
Gene uniquely mapped rate (%)	73.33	72.36	72.77	72.07	72.75	74.21

**Table 2:** 26 important pathways related to NK cell activation.

Pathway ID	KEGG Class	Pathways	NO. of DGEs	*p*-Value	q-Value
ko04622	Immune system	RIG-I-like receptor signaling pathway	21	2.67 × 10^−7^	1.97 × 10^−5^
ko05200	Cancers	Pathways in cancer	74	3.25 × 10^−7^	1.97 × 10^−5^
ko04015	Signal transduction	Rap1 signaling pathway	35	4.38 × 10^−7^	2.22 × 10^−5^
ko04392	Signal transduction	Hippo signaling pathway—multiple species	12	2.29 × 10^−6^	9.94 × 10^−5^
ko04390	Signal transduction	Hippo signaling pathway	30	8.82 × 10^−6^	2.98 × 10^−4^
ko04630	Signal transduction	Jak-STAT signaling pathway	31	1.86 × 10^−5^	5.13 × 10^−4^
ko04391	Signal transduction	Hippo signaling pathway -fly	17	6.36 × 10^−5^	1.21 × 10^−3^
ko04623	Immune system	Cytosolic DNA-sensing pathway	16	8.62 × 10^−5^	1.54 × 10^−3^
ko04217	Cell growth and death	Necroptosis	27	0.000362	6.12 × 10^−3^
ko04062	Immune system	Chemokine signaling pathway	30	0.000887	1.05 × 10^−2^
ko04610	Immune system	Complement and coagulation cascades	13	0.002281	2.04 × 10^−2^
ko04022	Signal transduction	cGMP—PKG signaling pathway	25	0.003715	3.05 × 10^−2^
ko04621	Immune system	NOD-like receptor signaling pathway	24	0.00501	3.81 × 10^−2^
ko04151	Signal transduction	PI3K-Akt signaling pathway	35	0.007833	5.18 × 10^−2^
ko04010	Signal transduction	MAPK signaling pathway	33	0.009703	6.02 × 10^−2^
ko04014	Signal transduction	Ras signaling pathway	27	0.010592	6.31 × 10^−2^
ko04350	Signal transduction	TGF-β signaling pathway	14	0.010813	6.32 × 10^−2^
ko04068	Signal transduction	FoxO signaling pathway	19	0.014861	8.07 × 10^−2^
ko04620	Immune system	Toll-like receptor signaling pathway	16	0.014868	8.07 × 10^−2^
ko04371	Signal transduction	Apelin signaling pathway	19	0.016059	8.46 × 10^−2^
ko04650	Immune system	Natural killer cell mediated cytotoxicity	17	0.018165	9.14 × 10^−2^
ko04310	Signal transduction	Wnt signaling pathway	21	0.018753	9.14 × 10^−2^
ko04670	Immune system	Leukocyte transendothelial migration	15	0.026129	1.24 × 10^−1^
ko04666	Immune system	Fc γ R-mediated phagocytosis	14	0.03014	1.39 × 10^−1^
ko04115	Cell growth and death	p53 signaling pathway	11	0.032294	1.47 × 10^−1^
ko05225	Cancers	Hepatocellular carcinoma	21	0.037815	1.62 × 10^−1^

## Data Availability

Raw sequencing data can be accessed in the NCBI SRA database under the accession number PRJNA859425.

## Supplementary Materials

The following supporting information can be downloaded at: https://www.mdpi.com/article/10.3390/genes14040888/s1, Table S1: forward and reverse primers used for qRT-PCR; Table S2: gene expression matrix of NDV and NC groups; Table S3: differentially expressed genes identified between NDV and NC groups; Table S4: gene ontology enrichment analysis for the DEGs; Table S5: KEGG pathway enrichment analysis for the DEGs; Table S6: NK cell activation related genes; Table S7: Kaplan-Meier overall survival analysis results for the selected 17 genes.
